# Comparison of the Spo_2_/Fio_2_ Ratio and the Pao_2_/Fio_2_ Ratio in Patients With Acute Lung Injury or Acute Respiratory Distress Syndrome

**DOI:** 10.15171/jcvtr.2014.06

**Published:** 2015-03-29

**Authors:** Nemat Bilan, Azar Dastranji, Afshin Ghalehgolab Behbahani

**Affiliations:** Pediatric Health Research Center, Tabriz University of Medical Sciences, Tabriz, Iran

**Keywords:** ARDS, ALI, Pao_2_/Fio_2_, Pulse Oximetry

## Abstract

***Introduction:*** Diagnostic criteria for acute lung injury (ALI) and Acute Respiratory Distress syndrome (ARDS) includes acute onset of disease, chest radiograph demonstrating bilateral pulmonary infiltrates, lack of significant left ventricular dysfunction and Pao_2_/Fio_2_ (PF) ratio ≤300 for ALI or ≤200 for ARDS. Recent criteria require invasive arterial sampling. The pulse oximetric saturation Spo_2_/Fio_2_ (SF) ratio may be a reliable non-invasive alternative to the PF ratio.

***Methods:*** In this cross-sectional study, we enrolled 70 patients with ALI or ARDS who were admitted in Tabriz children’s hospital pediatrics intensive care unit (PICU). Spo_2_, Fio_2_, Pao_2_, charted within 5 minutes of each other and calculated SF and PF were recorded to determine the relationship between SF and PF ratio. SF values were examined as a substitute of PF ratio for diagnosis ARDS and ALI.

***Results:*** The relationship between SF and PF ratio was described by the following regression equation: SF=57+0.61 PF (P<0.001). SF ratios of 181 and 235 corresponded of PF ratio 300 and 200. The SF cutoff of 235 had 57% sensitivity and 100% specificity for diagnosis of ALI. The SF cutoff of 181 had 71% sensitivity and 82% specificity for diagnosis of ARDS.

***Conclusion:*** SF ratio is a reliable noninvasive surrogate for PF ratio to identify children with ALI or ARDS with the advantage of replacing invasive arterial blood sampling by non-invasive pulse oximetry.

## Introduction

Acute lung injury (ALI) and acute respiratory distress syndrome (ARDS) are grave syndromes associated with high mortality and morbidity.^1,2^ It is estimated that 30% to 60% of all patients admitted to pediatric intensive care unit (PICU) require mechanical ventilation, and of these patients up to 25% suffer ALI and 5% to 10% have ARDS. With the implementation of lung-protective ventilation strategies, overall morbidity and mortality have improved significantly for both adult and children with ALI and ARDS.^3,4^ Based on American European Consensus Conference (AECC) in 1994, diagnostic criteria for ALI and ARDS require acute onset of disease, chest radiograph demonstrating bilateral pulmonary infiltrates, lack of significant left ventricular dysfunction and arterial partial pressure of carbon dioxid/Fraction of inspiratory oxygen (Pao_2_/Fio_2_) (PF) ratio ≤300 for ALI or ≤200 for ARDS.^5^ The first three components can be established with clinical history or noninvasively tools such as chest radiograph or echocardiography. However PF criteria require arterial blood sampling.^6,7^ Concerns about anemia following repeated blood sampling and tendency to implement less invasive approaches have led to less frequent blood gas measurements in critically ill patients.^8-9^ However studies in ARDS and ALI patients are lacking. Furthermore SF threshold values could be used for diagnosing ARDS and ALI.^6-10^

Pulse oximetry is the most commonly utilized technique to monitor oxygenation which is non-invasive and safe. In this method arterial hemoglobin O_2_ saturation is measured by differentiating oxy hemoglobin form deoxygenated hemoglobin using their respective light absorption at wave lengths of 660 nm (red) and 940 nm (infra red).^11,12^

Pulse oximetry is used for detection of hypoxia, prevention of hyperoxia, weaning from mechanical ventilation, and for titration of Fio_2_.^9-13^

In most PICUs daily arterial blood sampling to calculate the PF ratio is not feasible. Calculation of SF ratio as a surrogate for PF ratio for diagnose ARDS or ALI, which is less-invasive, is desirable.^14^ SF ratio determines the degree of hypoxemia non invasively without the need for arterial blood sampling.^7^

In this study, we examined the relationship between SF and PF ratio in critically ill patients with ALI and ARDS. It is presumed that the more available and less invasive SF ratio measurement can replace PF ratio measurement in the diagnosis of ALI and ARDS.

## Materials and methods

In this cross-sectional study 70 children with ARDS or ALI who were admitted in Tabriz children’s hospital PICU, Iran between 2012 and 2013 were enrolled. In patients with ARDS or ALI who were intubated and under mechanical ventilation with same Fio_2_; Pao_2_ was measured with arterial blood sampling and Spo_2_ was measured with pulse oximetry and charted within 5 min. SF and PF ratio were then calculated.

Inclusion criteria were children with ARDS or ALI and acute onset of disease and chest radiograph demonstrating bilateral pulmonary infiltrates , consistent with pulmonary edema.

Exclusion criteria were children with pulmonary edema due to heart failure and congenital heart disease and anatomic anomalies of lung or air ways and hypotension and weak pulses.

### Statistical analysis

Statistical analyses were performed using the Statistical Package for Social Sciences, version 17.0 (SPSS, Chicago, Illinois). Quantitative data were presented as mean ± standard deviation (SD), while qualitative data were demonstrated as frequency and percent (%). The categorical parameters were compared by χ^2^ tests, and the continuous variables were compared by independent *t* test. A P <0.05 was considered statistically significant. Relationship between SF and PF, described by linear regression equation. ROC curves were plotted to determine the sensitivity and specificity of the SF threshold values correlating with PF of 200 (ARDS) and 300 (ALI).

## Results

Of 70 children enrolled in this study, 38 patients were female (54.3%) and 32 patients were male (45.7%) . Mean age of study population was 32+ 5 months (minimum 2 and maximum 144 months).

A total of 70 data pairs 56 (80%) met the PF ratio criteria for ARDS and 14 (20%) met the PF criteria for ALI. The median time difference between charted values of Spo_2_ and Pao_2_ pairs was 5 minutes.

Table 1 demonstrates baseline findings of the patients enrolled in the study.

**Table 1:** Base line findings of the patients

	Max-Min	Mean±SD
Pao_2_/ Fio_2_	298-46	155±61
Spo_2_/Fio_2_	248-77	152±47
Spo_2_	99-71	94±4
Fio_2_	100-40	67±18
Pao_2_	176-41	96±25
Age	144-2	32±5

Age had no significant relationship with either SF ratio (P = 0.81) or PF ratio (P =0.99). Similarly, gender did not have a significant relationship with either SF ratio (P = 0.77) or PF ratio (P =0.06).

**Figure 1:**
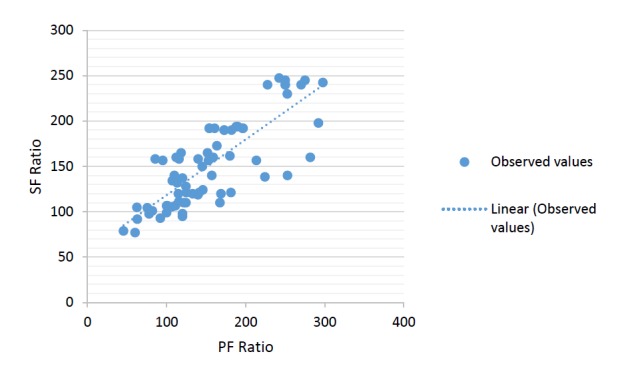
S/F ratio vs P/F ratio scatter plot for the derivation data set. The line represents the best fit linear relationship SF=57+0.61PF (P<0.001).

In general, SF ratio could be predicted well from PF ratio, described by the linear regression equation (SF =57+0.61 PF). Based on this equation a PF ratio of 300 corresponds to an SF ratio of 235 and PF ratio of 200 to an SF ratio of 181 [P <0.001 (Figure 1)]. The SF cut off of 235 had 57% sensitivity and 100% specificity for the diagnosis of ALI and cut off of 181 had 71% sensitivity and 82% specificity for the diagnosis of ARDS.

**Figure 2:**
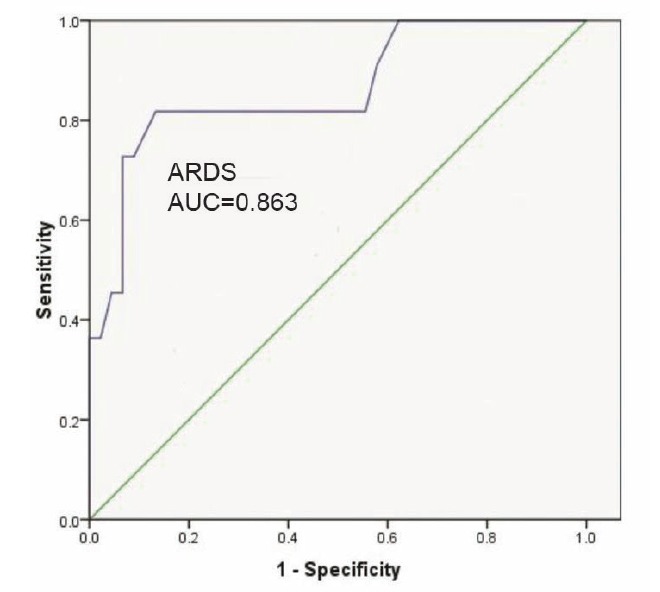
ROC curves for S/F vs P/F ratios of ≤200 (ARDS).

**Figure 3:**
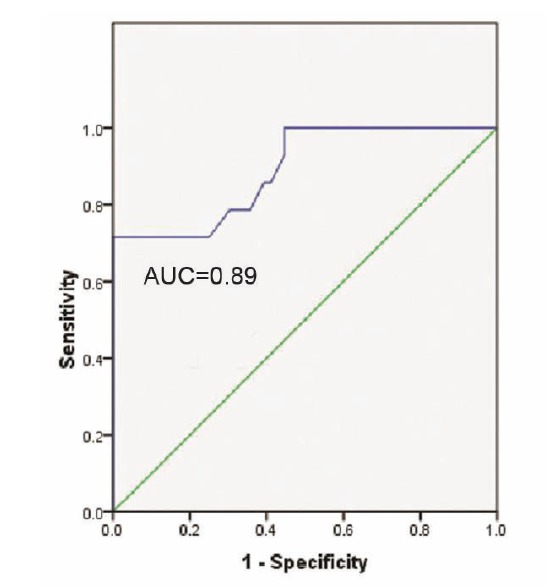
ROC curves for S/F vs. P/F ratios of ≤ 200 (ARDS) and S/F vs. P/F ratios of ≤ 300 (ALI) for the derivation data set.

SF ratio had excellent discrimination ability for ARDS (AUC=0.86) (Figure 2) and good discrimination ability for ALI and ARDS (AUC=0.89) (Figure 3).

## Discussion

ALI and ARDS are major contributors to morbidity and mortality for patients admitted to PICU.^15^ The routine use of pulse oximetry and capnography have led to less frequent arterial blood gas sampling. In most PICUs, pulse oximetry is readily available and is being used routinely for continuous monitoring of oxygenation status.^16-18^ Pulse oximetry circumvents the harms and costs of arterial blood sampling.^19^ Using SF ratio for diagnosing ALI and ARDS leads to identification of undiagnosed cases of aforementioned syndromes.^20^

SF ratio may be useful in many organ failure scores, such as lung injury scores^21^, multi organ dysfunction score^22^, sequential organ failure assessment^23^, as an alternative to PF ratio to estimate the degree of hypoxemia.

In this study we enrolled 70 patients with ALI or ARDS. Pao_2_ and Spo_2_ were measured with the same Fio_2_ and SF and PF ratio were calculated. The relationship between SF and PF ratio was described with the following equation: SF=57+0.61 PF. SF ratio threshold values for ALI was 235 and for ARDS was 181 corresponding to PF ratio 300 and 200.

A similar study was conducted by Khemani et al.^24^ on pediatric population. They report that a cut-off of 201 for SF could predict PF for ARDS with 84% sensitivity and 78% specificity and a cut-off of 263 for SF could predict ALI with 93% sensitivity and 43% specificity. However, they didn’t have age limitation. Also the time interval between pulse oximetry and arterial blood sampling was 15 minutes which is longer that our study. They did not examine the relationship between SF and PF ratio with sex and gender.

In adult patients, Rice et al.^25^ report that a cut-off 235 for SF could predict ARDS with 85% sensitivity and 85% specificity and cut-off of 315 for SF could predict ALI with 91% sensitivity and 56 % specificity. In this study, we examined the relationship between age and gender with PF and SF ratio. We measured Pao_2_ and Spo_2_ in maximum 5 minutes apart. The SF ratio thresholds determined in this study were based on the PF ratio which is proposed by the AECC.

There are limitations to this study. First, ABG and pulse oximetry measurements were not simultaneous. Given that changes in Spo_2_ and Pao_2_ can take place quickly, this could affect our results. In addition we did not control for PH, hemoglobin, Paco_2_, body temperature, ventilator set up. These factors also could influence the relationship between Spo_2_and Pao_2_.

## Ethical issues

The study was approved by the local Ethics Committee.

## Competing interests

Authors declare no conflict of interests in this study.

## Conclusion

According to this study SF ratio is a reliable non invasive and readily available marker for PF ratio for the diagnosis of children with ALI or ARDS which replaces arterial blood sampling by pulse oximetry. Considering complications of arterial blood sampling such as anemia, and bleeding in critical care patients, pulse oximetry is a desirable replacement for arterial blood sampling.

## References

[1] Ware LB, Matthay MA (2000). The acute respiratory distress syndrome. N Engl J Med.

[2] Rubenfeld GD, Caldwell E, Peabody E (2005). Incidence and outcomes of acute lung injury. N Engl J Med.

[3] Bernard GR, Artigas A, Brigham KL (1994). The American-European Consensus Conference on ARDS: definitions,mechanisms, relevant outcomes, and clinical trial coordination. Am J Respir Crit Care Med.

[4] Merlani P, Garnerin P, Diby M, Ferring M (2001). Quality improvement report: linking guideline to regular feedback to increase appropriate requests for clinical test; blood gas analysis in intensive care. BMJ.

[5] Bernard GR, Artigas A, Brigham KL, Carlet J, Falke K, Hudson L (1994). Thoracic SoG-The American European Consensus Conference on ARDS efinitions, mechanisms. Am J Respir Crit Care Med.

[6] Pilon CS, Leathley DM, London R (1997). Practice guideline for arterial blood gas measurement in the intensive care unit decreases numbers and increases appropriateness of tests. Crit Care Med.

[7] Jensen LA, Onyskiw JE, Prasad NG (1998). Meta-analysis of arterial oxygen saturation monitoring by pulse oximetry in adults. Heart Lung.

[8] Ferguson ND, Frutos-Vivar F, Esteban A, Fernández-Segoviano P, Aramburu JA, Nájera L (2005). Acute respiratory distress syndrome: underrecognition by clinicians and diagnostic accuracy of three clinical definitions. Crit Care Med.

[9] Roberts D, Ostryzniuk P, Loewen E, Shanks A, Wasyluk T, Pronger L (1991). Control of blood gas measurements in intensive-care units. Lancet.

[10] Perkins GD, McAuley DF, Giles S (2003). Do changes in pulse oximeter oxygen saturation predict equivalent changes in arterial oxygen saturation?. Crit Care.

[11] Ms Mortz. US patent 714,803,2004 pulse oximeter probe off detection system.

[12] Kliegman RM, Stanton BM, Geme JS, Schor N, Behrman RE. Nelson Textbook of Pediatrics.19 th ed. Elsevier; 2011. p.318.

[13] Slutsky AS (1993). Mechanical ventilation American College of Chest Physicians’ Consensus Conference. Chest.

[14] Pandharipande PP1, Shintani AK, Hagerman HE, St Jacques PJ, Rice TW, Sanders NW (2009). Derivation and validation of Spo2/Fio2 ratio to impute for Pao2/Fio2 ratio in the respiratory component of the Sequential Organ Failure Assessment score. Crit Care Med.

[15] Khemani RG, Markovitz BP, Curley MAQ (2007). Epidemiologic factors of mechanically ventilated PICU patients in the United States [abstract]. Pediatr Crit Care Med.

[16] Numa AH, Newth CJ (1995). Assessment of lung function in the intensive care unit. Pediatr Pulmonol.

[17] Montgomery AB, Stager MA, Carrico CJ (1985). Causes of mortality in patients with the adult respiratory distress syndrome. Am Rev Respir Dis.

[18] Jubran A (2004). Pulse oximetry. Intensive Care Med.

[19] Jubran A, Tobin MJ (1990). Reliability of pulse oximetry in titrating supplemental oxygen therapy in ventilator-dependent patients. Chest.

[20] (2000). Ventilation with lower tidal volumes as compared with traditional tidal volumes for acute lung injury and the acute respiratory distress syndrome: the Acute Respiratory Distress Syndrome Network. N Engl J Med.

[21] Murray JF, Matthay MA, Luce JM, Flick MR (1988). An expanded definition of the adult respiratory distress syndrome. Am Rev Respir Dis.

[22] Marshall JC, Cook DJ, Christou NV, Bernard GR, Sprung CL, Sibbald WJ (1995). Multiple organ dysfunction score: a reliable descriptor of a complex clinical outcome. Crit Care Med.

[23] Le Gall JR, Lemeshow S, Saulnier F (1993). A new Simplified Acute Physiology Score (SAPS II) based on a European/North American multicenter study. JAMA.

[24] Khemani RG, Patel NR, Bart RD 3rd, Newth CJ (2008). Comparison of the pulse oximetric saturation/fraction of inspired oxygen ratio and the PaO2/fraction of inspired oxygen ratio in children. Chest.

[25] Rice TW, Wheeler AP, Bernard GR, Hayden DL, Schoenfeld DA, Ware LB (2007). Comparison of the SpO2/FIO2 ratio and the PaO2/FIO2 ratio in patients with acute lung injury or ARDS. Chest.

